# Screening Linear and Circular RNA Transcripts from Stress Granules

**DOI:** 10.1016/j.gpb.2022.01.003

**Published:** 2022-01-25

**Authors:** Shuai Chen, Jinyang Zhang, Fangqing Zhao

**Affiliations:** 1 Beijing Institutes of Life Science, Chinese Academy of Sciences, Beijing 100101, China; 2 University of Chinese Academy of Sciences, Beijing 100049, China; 3 Center for Excellence in Animal Evolution and Genetics, Chinese Academy of Sciences, Kunming 650223, China; 4 Key Laboratory of Systems Biology, Hangzhou Institute for Advanced Study, University of Chinese Academy of Sciences, Hangzhou 310013, China

**Keywords:** Stress granule, Circular RNA, Protein–RNA interaction, RNA-seq, Hepatocellular carcinoma

## Abstract

**Stress granules** (SGs) are cytoplasmic ribonucleoprotein assemblies formed under stress conditions and are related to various biological processes and human diseases. Previous studies have reported the regulatory role of some proteins and linear RNAs in SG assembly. However, the relationship between **circular RNAs** (circRNAs) and SGs has not been discovered. Here, we screened both linear RNAs and circRNAs in SGs using improved total RNA sequencing of purified SG cores in mammalian cells and identified circular transcripts specifically localized in SGs. circRNAs with higher SG-related RNA-binding protein (RBP) binding abilities are more likely to be enriched in SGs. Furthermore, some SG-enriched circRNAs are differentially expressed in **hepatocellular carcinoma** (HCC) and adjacent tissues. These results suggest the regulatory role of circRNAs in SG formation and provide insights into the biological function of circRNAs and SGs in HCC.

## Introduction

Stress granules (SGs) are membrane-less condensates that are dynamically and reversibly formed with ribonucleoproteins by phase separation [1]. SGs usually assemble in response to stress conditions [2]. Recent studies have shown that SGs are associated with cellular biological processes and various human diseases, such as degenerative diseases and malignant tumors [3], [4], [5]. Some RNA-binding proteins (RBPs) like Ras GTPase-activating protein-binding protein (G3BP), TIA-1, and TAR are proved to be essential in SG assembly, and G3BP1 serves as the core component of SGs [6], [7], [8]. In addition to proteins, RNAs are associated with SG formation as well. A fraction of translationally proceeded or arrested mRNAs is localized in SGs [9], [10]. Moreover, the principle of mRNA and non-coding RNA (ncRNA) accumulation in SGs is revealed in the transcriptome of SG cores [11]. However, these studied SG-related RNAs are all linear transcripts. Circular RNAs (circRNAs) specifically recruited to SGs have not been elucidated.

To address this issue, we improved the purification and sequencing approach of total RNA in SGs and screened circRNAs in SGs from the transcriptome of purified SG cores induced from mammalian cells. We identified 130 SG-enriched circRNAs and 2462 SG-depleted circRNAs, which revealed the specific accumulation of circRNAs in SGs. The circRNAs enriched in SGs exhibited stronger ability to interact with SG-related RBPs, even the SG core component G3BP2. In addition, some SG-enriched circRNAs were differentially expressed in hepatocellular carcinoma (HCC) and adjacent tissues, indicating the potential role of circRNAs and SGs in HCC.

## Results

### RNA transcripts enriched in SG cores

**Figure 1:**
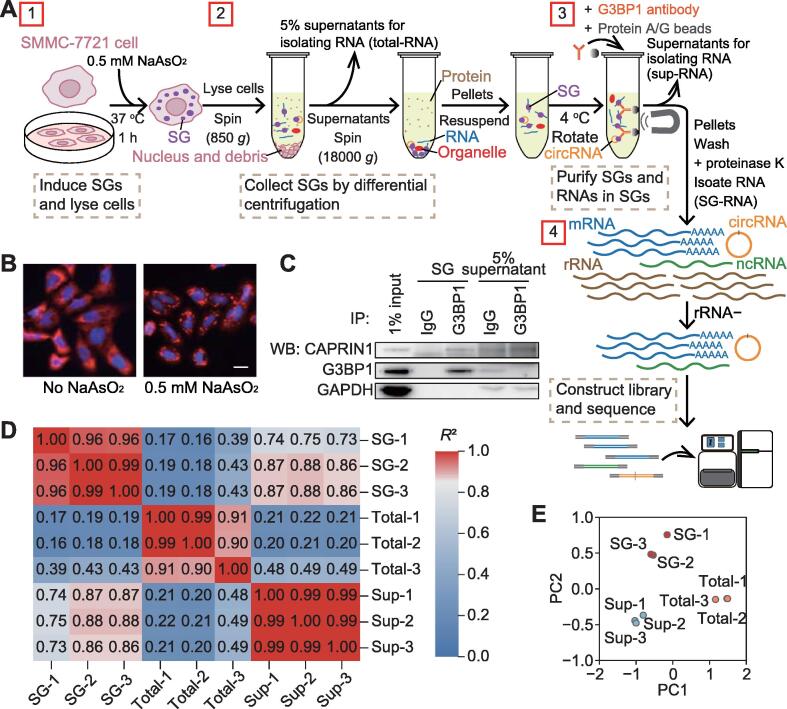
**Screening SG-specific transcripts in SGs of SMMC-7721 cells** **A.** The schematic workflow of inducing, isolating, and purifying SGs and sequencing of RNAs in SGs. Total-RNA indicates the RNA from cell lysates (excluding nucleus and debris); SG-RNA indicates the RNA from purified SGs; and sup-RNA indicates the RNA from the supernatants without SGs of the IP solution. **B.** Immunofluorescence of SGs in control and NaAsO_2_-stimulated cells. Scale bar, 20 μm. **C.** Western blot of SG core protein G3BP1 in purified SGs and supernatants. **D.** Pairwise Pearson correlation coefficients between replicates of SG-RNA, total-RNA, and sup-RNA. **E**. PCA of individual replicates of SG-RNA, total-RNA, and sup-RNA. SG, stress granule; IP, immunoprecipitation; WB, Western blot; PCA, principal component analysis; PC, principal component.

To determine the transcriptome of SG cores, we isolated total RNA from purified SG cores in triplicates from SMMC-7721 cells, followed by RNA sequencing (RNA-seq) analysis (Figure 1A). Since only few SGs formed in cells grown under normal conditions, we induced SGs by incubating cells with 0.5 mM NaAsO_2_ for 1 h. Immunofluorescence analysis of G3BP1 protein identified cytoplasmic SG condensates after stimulation (Figure 1B). Then, SGs were separated from cell lysates by differential centrifugation and purified by immunoprecipitation (IP) with G3BP1-specific antibodies. Western blot analysis of IP products showed that the SG core component G3BP1 and another SG-associated protein CAPRIN1 could be enriched by specific antibodies (Figure 1C). For each sample, RNA was isolated for RNA-seq from purified SGs, 5% of cell lysates, and the supernatants without SGs of the IP solution, which are referred to as SG-RNA, total-RNA, and sup-RNA, respectively (Figure 1A).

Pairwise correlation analysis of the transcriptomes of the three RNA groups demonstrated the high reproducibility between experimental replicates in the same group (*R*^2^ > 0.9; Figure 1D). Moreover, both SG-RNA and sup-RNA transcriptomes showed low correlations with total-RNA transcriptomes (*R*^2^ < 0.5), suggesting that SG-RNA transcriptomes are different from total-RNA transcriptomes. In addition, principal component analysis (PCA) also highlighted the similarities within each transcriptome triplicates of SG-RNA, total-RNA, and sup-RNA as well as the differences between these three RNA groups (Figure 1E). Therefore, these findings demonstrate that SGs contain specific transcriptomes compared with cytosolic total RNA and the RNAs outside of SGs.

### SG-enriched RNAs are longer and have higher GC content

**Figure 2:**
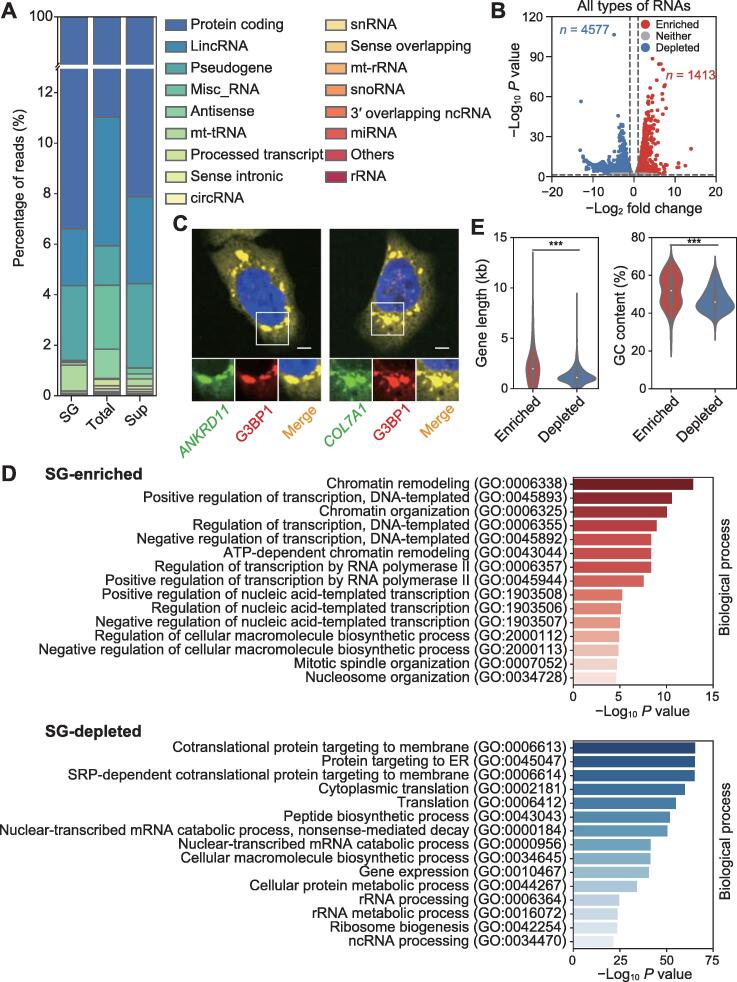
**Characterization of all types of RNAs in SGs** **A.** Composition of the transcriptomes of SG-RNA, total-RNA, and sup-RNA groups. **B.** Volcano plot depicting abundance of all types of transcripts in SG-RNA *versus* total-RNA. Red and blue dots indicate RNAs that are significantly enriched or depleted in SGs, respectively. **C.** RNA FISH validation of RNAs enriched in SGs (*ANKRD11* and *COL7A1*). Scale bar, 5 μm. **D.** GO analysis for SG-enriched and SG-depleted RNAs. **E.** and **F.** Violin plots depicting the difference in transcript length (E) and GC content (F) between all types of SG-enriched and SG-depleted RNAs. ***, *P* < 0.001. LincRNA, long intergenic non-coding RNA; misc_RNA, miscellaneous RNA; mt-tRNA, mitochondrial transfer RNA; circRNA, circular RNA; snRNA, small nuclear RNA; mt-rRNA, mitochondrial ribosomal RNA; snoRNA, small nucleolar RNA; miRNA, microRNA; rRNA, ribosomal RNA; FISH, fluorescence *in situ* hybridization; GO, Gene Ontology.

Since there were differences between the transcriptomes of SG-RNA, sup-RNA, and total-RNA, we thought to identify the specific transcripts that are enriched or depleted in SGs. According to the general analysis of RNA profiles in these transcriptomes, more fraction of mRNA transcripts was detected in SG-RNA than in total-RNA and sup-RNA, while long non-coding RNA (lncRNA) and circRNA transcripts were fewer (Figure 2A; Table S1), indicating the translation regulation roles of SGs. Furthermore, differential expression analysis indicated that 1413 transcripts were significantly enriched in SGs, while 4577 transcripts were significantly depleted in SGs compared with the total-RNA group (adjusted *P* < 0.05; Figure 2B). RNA fluorescence *in situ* hybridization (FISH) of two SG-enriched genes, *ANKRD11* and *COL7A1*, together with immunofluorescence showed that both mRNAs were colocalized with SGs (Figure 2C). Gene Ontology (GO) analysis for SG-enriched and SG-depleted transcripts showed that the SG-enriched transcripts were involved in regulating RNA transcription, while the SG-depleted transcripts were associated with membrane targeting of proteins (Figure 2D). These findings demonstrated the transcription regulation role of SGs, which was supported by a previous study [12], as transcription of most genes is inhibited under stress conditions. We also examined the molecular features of RNAs in SGs. The SG-enriched RNAs tended to be longer (2267 nt *versus* 1252 nt on average) and had higher GC content than SG-depleted RNAs (Figure 2E and F).

### SG-enriched circRNAs have stronger ability to bind SG-related RBPs

**Figure 3:**
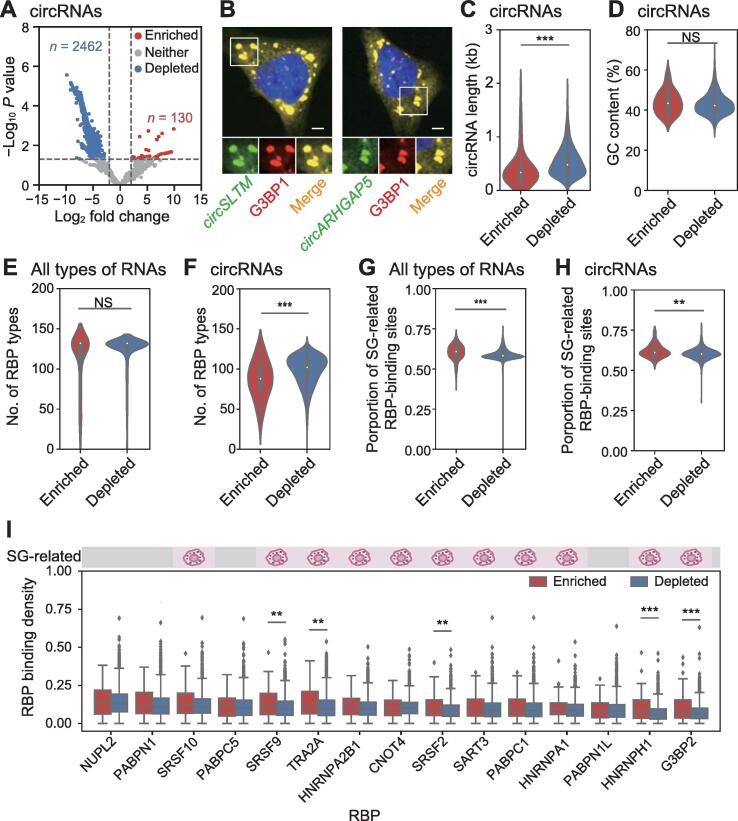
**Characterization of circRNAsenrichedin SGs** **A.** Volcano plot showing circRNA abundance in SG-RNA *versus* total-RNA. Red and blue dots indicate circRNAs that are significantly enriched or depleted in SGs, respectively. *P* values were calculated using GLM test in edgeR. **B.** RNA FISH validation of circRNAs enriched in SGs (*circSLTM* and *circARHGAP5*). Scale bar, 5 μm. **C.**–**H.** Violin plots showing the difference in transcript length (C), GC content (D), number of RBP types (F), and proportion of SG-related RBP-binding sites (H) between SG-enriched and SG-depleted circRNAs, as well as the number of RBP types (E) and proportion of SG-related RBP-binding sites (G) between all types of SG-enriched and SG-depleted RNAs. **I.** Box plot showing the binding density of top 15 RBPs with higher binding ability to SG-enriched circRNAs. The symbols on the top shows whether these RBPs are targeted to SGs. **, *P* < 0.01; ***, *P* < 0.001; NS, not significant.

As increasing studies have highlighted the importance of circRNAs in various biological processes, we further used the CIRI software [13], [14], [15], [16] to identify and quantify the circRNAs that were recruited to SGs. According to the differential expression analysis, 130 circRNAs were significantly enriched in SGs, while 2462 were significantly depleted in SGs, compared to those in the total-RNA group (*P* < 0.05) (Figure 3A). Among the enriched circRNAs, 66 have the sequence and annotation information in the circAtlas database [17], [18] (Table S2). We also validated the localization of two circRNAs, *circSLTM* and *circARHGAP5*, in SGs by RNA FISH and immunofluorescence (Figure 3B).

To uncover the characteristics of enriched circRNAs in SGs, we performed analysis on the sequence features and molecular functions of these circRNAs. Although longer mRNA transcripts tended to be enriched in SGs (Figure 2E), SG-enriched circRNAs (average length = 413 nt) were significantly shorter than SG-depleted circRNAs (average length = 537 nt) (*P* < 0.001, Wilcoxon test) (Figure 3C). In addition, there was no significant difference in GC content between SG-enriched and SG-depleted circRNAs (Figure 3D), indicating that GC content is not associated with SG accumulation of circRNAs. Given that RNAs that bind more SG-related RBPs are more likely to be localized into SGs, whether SG-enriched RNAs exhibit higher RBP binding ability were evaluated. RBP-binding sites prediction by RBPmap web server [19] found that there was no significant difference in RBP types between SG-enriched RNAs and SG-depleted RNAs (Figure 3E). However, SG-enriched circRNAs could bind fewer types of RBPs than SG-depleted circRNAs (Figure 3F). Surprisingly, we observed that the proportion of binding sites of SG-related RBPs were higher in SG-enriched RNAs than in SG-depleted RNAs (Figure 3G), as well as higher in SG-enriched circRNAs than in SG-depleted circRNAs (Figure 3H). Furthermore, analysis of the top 15 RBPs with higher binding density on SG-enriched circRNAs showed that most of them were SG-related RBPs (Figure 3I). Among them, G3BP2 was a core component of SGs, indicating the role of circRNAs in regulating SG formation. Taken together, these results demonstrate that circRNAs with higher capability of binding SG-related RBPs show a stronger preference to be recruited into SGs and circRNAs are involved in the formation of SGs through binding to their component proteins.

### Differentially expressed SG-enriched circRNAs in HCC

Previous studies demonstrate that SGs are associated with human diseases like cancer [20]. We further examined whether there are SGs-enriched circRNAs associated with HCC, since the SG transcriptomes were purified from HCC cells. We screened 35 SG-enriched circRNAs that were expressed in HCC and adjacent tissues, and found three circRNAs, *circEXOC6B*, *circCALD1*, and *circVAMP3* that were significantly down-regulated in HCC tissues (Figure S1). These results indicate the potential roles of mutual interplay between these circRNAs and SGs in HCC.

## Discussion

Assembly of SGs can be influenced by stress stimulation, as well as regulated by proteins and RNAs. RBPs are reported to play important roles in the formation of SGs [2], while RNAs are thought to act as scaffolds to bind RBPs and thus to regulate SG assembly. It has been reported that GIRGL lncRNA interacts with CAPRIN1 to drive SG formation [21]. As SG-enriched circRNAs exhibit higher ability of binding SG-related RBPs, *e.g.*, G3BP2, these circRNAs are presumed to be related to the formation of SGs. Although it has been assumed that RNAs can be recruited to SGs through the SG-related RBPs, there is no direct evidence to support this hypothesis. Moreover, RNA translation inhibition is also reported to be relevant to SG assembly [22]. However, no translation potential was detected in SG-enriched circRNAs (Table S2), suggesting that the localization of circRNAs in SGs should not be due to translation. Although longer RNAs are more likely to be enriched in SGs, we observed that SG-enriched circRNAs are shorter than SG-depleted circRNAs. According to our previous study, most of circRNAs are less than 600 nt in length [23], [24]. Considering that the length difference between SG-enriched and SG-depleted circRNAs is limited, it may have little effect on the SG-related RBP binding of circRNAs. It should be noted that as SGs are dynamic condensates, high speed of centrifugation may affect the stability of SGs. Some SG components with weak interactions may be missed by differential centrifugation without crosslinking. However, the internal core components in SGs are more stable [25], which can be effectively captured using the approach developed in this study.

In summary, our study provides novel insights into SG-related circRNAs through transcriptome analysis of SG cores. The ability of binding SG-related RBPs of circRNAs is related to their localization in SGs, suggesting the role of circRNAs in SG formation. Also, some SG-enriched circRNAs are differentially expressed between HCC and adjacent tissues. Further studies are still needed to elucidate the specific mechanisms and cellular consequences of circRNAs in SG formation.

## Materials and methods

### Cell culture and stress conditions

HCC SMMC-7721 cells were cultured in the cell culture medium containing Dulbecco’s modified Eagle’s medium (DMEM; Catalog No. C11995500BT, Gibco, Carlsbad, CA), 10% fetal bovine serum (FBS; Catalog No. 10270-106, Gibco), and 1% penicillin–streptomycin (Catalog No. 15140-122, Gibco) at 37 °C and 5% CO_2_. For stress experiments, cells were treated with 0.5 mM NaAsO_2_ (Catalog No. S7400-100g, Sigma-Aldrich, Saint Louis, MO) for 1 h at 37 °C and 5% CO_2_.

### Isolation of SGs

Isolation of SGs was performed as described in a previous study [26]. SMMC-7721 cells were seeded on 15-cm culture dishes (Catalog No. 430599, Corning, Cambridge, MA) and grown to 80% confluence. Cell culture medium was exchanged with fresh culture medium 1 h prior to the stress treatment. After treating with NaASO_2_, cells were washed once with 1× PBS (137 mM NaCl, 2.7 mM KCl, 10 mM Na_2_HPO_4_, 2 mM KH_2_PO_4_, and pH 7.4), collected by scraping and pelleted by centrifugalization at 1500 *g* for 3 min. Cells were re-suspended in 1 ml SG lysis buffer [50 mM Tris-HCl pH 7.4, 2 mM MgOAc, 100 mM KOAc, 50 μg/ml Heparin, 0.5 mM DTT, 0.5% NP40, 1 U/μl Recombinant RNase Inhibitor (Catalog No. 2313A, Takara, Tokyo, Japan), and EDTA-free protease inhibitor cocktail tablets (Catalog No. 04693132001, Roche, Basel, Switzerland)] and passed through a 25G 5/8 needle 7 times to lyse cells. Cell debris were removed after centrifugalizing at 1000 *g* for 5 min at 4 °C. Then, 50 μl of the lysate supernatant was collected to isolate total RNA or perform Western blot. The remaining lysate was spun at 1,8000 *g* for 20 min at 4 °C to pellet SG cores. The pellets were re-suspended by SG lysis buffer and incubated with 25 μl protein A/G magnetic beads (Catalog No. 88802, ThermoFisher Scientific, Waltham, MA) twice for 30 min at 4 °C with gentle rotation to remove nonspecific binding proteins. Magnetic beads were removed using a magnet. The supernatants were incubated with G3BP1 antibodies (Catalog No. 13057-2-AP, Proteintech, Rosemont, IL) for 1 h at 4 °C followed by incubating with 25 μl protein A/G magnetic beads for 3 h at 4 °C with gentle rotation. The supernatants were collected to isolate supernatant RNA or perform Western blot. Beads were then washed three times with wash buffer 1 (20 mM Tris-HCl pH 8.0, 200 mM NaCl, and 1 U/μl Recombinant RNase Inhibitor), wash buffer 2 (20 mM Tris-HCl pH 8.0, 500 mM NaCl, and 1 U/μl Recombinant RNase Inhibitor), and wash buffer 3 (SG lysis buffer and 2 M Urea). The beads were then eluted to acquire SG proteins or resuspended with 200 μl Proteinase K buffer (1× TE buffer, 2 M Urea, and 1 U/μl Recombinant RNase Inhibitor) for 15 min at 37 °C, and the supernatants were collected to isolate SG RNAs.

### RNA isolation, library construction, and RNA-seq

RNA was isolated using TRIzol LS reagent (Catalog No. 15596026, Invitrogen, Carlsbad, CA) according to the manufacturer’s protocol, and was dissolved in 10 μl RNase-free water. The concentration of RNA was assessed using Qubit (Catalog No. 2061412, ThermoFisher Scientific).

Ribosomal RNAs (rRNAs) were depleted using KAPA RiboErase Kit (Catalog No. 07962266001, KAPA biosystem, Wilmington, MA). cDNA libraries from 10 ng ribosomal-depleted RNA were prepared using KAPA RNA HyperPrep kit (Catalog No. 7962428001, KAPA biosystem) according to the manufacturer’s protocol. The cDNA libraries (3 from cell lysates, 3 from SGs, and 3 from supernatants without SGs) were sequenced on the Illumina NovaSeq 6000 platform with 150 bp pair-end reads.

### RNA FISH and immunofluorescence assay

RNA FISH was performed using the biotin-labeled RNA probes in the exon junction region of mRNAs and the back-splice region of circRNAs. The sense and antisense DNA oligos containing T7 promoters and probes were synthesized and annealed (Table S3). Biotin-labeled RNA probes were transcribed from the annealed products using a HiScribe T7 Quick High Yield RNA Synthesis Kit (Catalog No. E2050S, New England Biolabs, Ipswich, MA) and biotin RNA labeling mix (Catalog No. 11685597910, Roche) according to the manufacturers’ protocols. Cells were seeded on glass coverslips and grown to 60%–80% confluent. Cells were washed with 1× PBS, fixed with 4% paraformaldehyde, and permeabilized with 0.3% Triton-X 100. The prehybridization and hybridization experiments were performed using Fluorescent *In Situ* Hybridization Kit (Catalog No. C10910, Ribobio, Guangzhou, China) according to the manufacturer’s protocol. Signals were detected using FITC labeled anti-biotin antibody (Catalog No. ab6650, Abcam, Cambridge, UK).

For immunofluorescence assay, the RNA probe incubated cells were blocked with 5% bovine serum albumin (BSA; Catalog No. 36103ES25, YEASEN, Shanghai, China). Antibodies were diluted in 1× PBS containing 0.3% Triton-X 100 and 1% BSA. Cells were incubated with mouse anti-G3BP1 (Catalog No. 66486-1-Ig, Proteintech) primary antibody for 2 h at room temperature, followed by three times of washes with 1× PBS. Cells were then incubated with Alexa Fluor 555 goat anti-rabbit (Catalog No. 4413, Cell Signaling Technology, Beverly, MA) primary antibody for 2 h at room temperature, followed by three times of washes with 1× PBS. The nucleus was stained using Hochest33342. The cell slices were mounted and images were acquired using Olympus IX83 confocal microscope (Olympus, Tokyo, Japan).

### Western blot

Protein samples were separated by SDS–PAGE and transferred onto polyvinylidene fluoride membrane (Catalog No. ISEQ00010, Millipore, Billerica, MA). The membranes were blocked with 5% nonfat dried milk in TBST buffer (10 mM Tris-HCl, 150 mM NaCl, 0.1% Tween-20, and pH 7.6) for 1 h at room temperature and incubated with protein-specific primary antibodies for 1 h at room temperature, followed by three times of washes with TBST buffer. The membranes were then incubated with HRP-conjugated secondary antibodies, followed by three times of washes with TBST buffer. The detailed information for the primary and secondary antibodies used in this analysis is provided in Table S4. The protein signals were visualized using ECL chemiluminescence reagents (Catalog No. BE6706-100, EASYBIO, Beijing, China), and images were acquired using Tanon 5200 chemiluminescent imaging system (Tanon, Shanghai, China).

### Quality control of RNA-seq data

Quality control was performed using FastQC (v0.11.9), and results were aggregated and visualized using MultiQC (v1.7) [27]. Sequencing adapter and low-quality sequences were trimmed using Trim_galore (v.0.6.6) with ‘stringency 6’ parameter. The human rRNA sequences were downloaded from the NCBI Nucleotide database using keyword ““*Homo sapiens*”[Organism] AND biomol_rrna[PROP]”. Trimmed reads were aligned to rRNA sequences using bowtie2 (v2.3.4.3) [28] with ‘--very-sensitive’ parameter, and aligned reads were depleted using samtools (v1.10) [29].

### RNA-seq data analysis

The reference human genome and annotation (release 19, GRCh37.p13) were downloaded from GENCODE project. CIRI2 (v2.0.6) and CIRIquant (v1.1.2) were used for detection, quantification, and differential expression analysis of circRNAs in the RNA-seq libraries [14], [13]. Gene expression values were calculated using the HISAT2 (v2.1.0) and StringTie (v2.0.5) pipelines [30], [31]. Gene differential expression analysis was performed using the GLM model in edgeR (v.3.26.8) package [32]. PCA was performed using ‘sklearn.decomposition.PCA’ function in the scikit-learn package [33].

### circRNA characterization and RBP analysis

The full-length sequences of circRNAs were downloaded from the circAtlas (v2.0) database [17]. Only circRNAs that included in circAtlas database were kept for downstream analysis. The binding sites of human RPBs were predicted using the RBPmap (v1.2) webserver with high stringency level and conservation filter, and results were parsed using custom scripts [19]. The expression levles of circRNAs in HCC dataset were obtained from a previous study [13]. SG-related protein database was downloaded from PhaSepDB 2.0 [34]. GO analysis was calculated using Enrichr [35].

## Data availability

The raw sequence data in this study have been deposited in the Genome Sequence Archive for Human [36] at the National Genomics Data Center, Beijing Institute of Genomics, Chinese Academy of Sciences / China National Center for Bioinformation (GSA-Human: HRA001512), and are publicly accessible at https://ngdc.cncb.ac.cn/gsa-human/.

## Competing interests

The authors have declared no competing interests.

## CRediT authorship contribution statement

**Shuai Chen:** Conceptualization, Validation, Investigation, Writing – original draft. **Jinyang Zhang:** Formal analysis, Data curation, Writing – original draft. **Fangqing Zhao:** Conceptualization, Writing – review & editing, Supervision, Project administration, Funding acquisition. All authors have read and approved the final manuscript.

## Supplementary material

Supplementary Figure S1 **SG-enriched circRNAs in HCC and adjacent tissues**. *, *P* < 0.05; **, *P* < 0.01; ***, *P* < 0.001.
