# Supplementary figures and images for: Screening Linear and Circular RNA Transcripts from Stress Granules
Source: Genomics Proteomics Bioinformatics. 2022 Jan 25;21(4):886–93. doi: 10.1016/j.gpb.2022.01.003 (PMC10787114; doi:10.1016/j.gpb.2022.01.003)

**
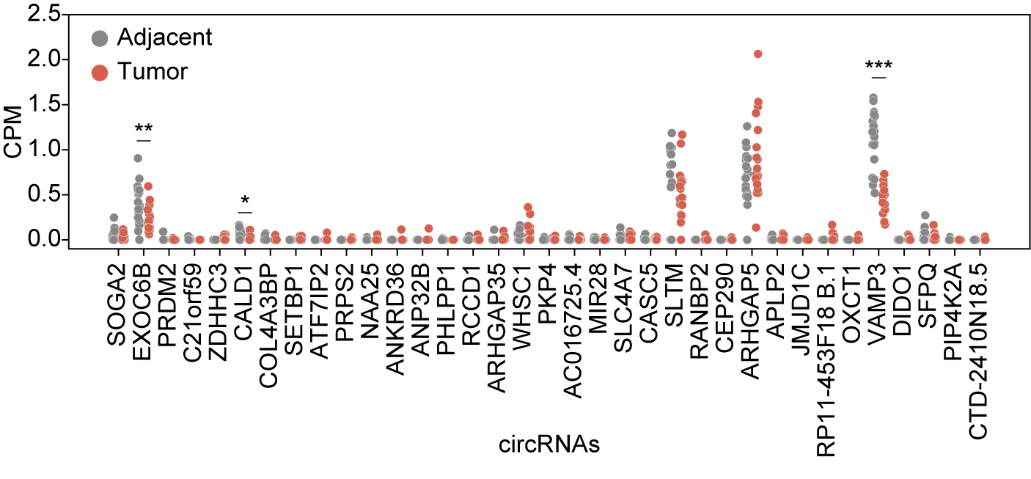
**

Supplement: Supplementary Figure S1 — SG-enriched circRNAs in HCC and adjacent tissues. *, P < 0.05; **, P < 0.01; ***, P < 0.001. [file mmc1.docx]
