# Supplementary material for: Screening Linear and Circular RNA Transcripts from Stress Granules
Source: Genomics Proteomics Bioinformatics. 2022 Jan 25;21(4):886–93. doi: 10.1016/j.gpb.2022.01.003 (PMC10787114; doi:10.1016/j.gpb.2022.01.003)
Supplement: Supplementary Table S1 [file mmc2.docx]

**Table S1 RNA composition, reads number, and gene number of individual replicates in the transcriptomes of SG-RNAs, total-RNAs, and sup-RNAs**

| **Gene type** | **Replicate** | **Reads number** | | |  | **Gene number** | | |
| --- | --- | --- | --- | --- | --- | --- | --- | --- |
|  |  | **SG** | **Total** | **Sup** |  | **SG** | **Total** | **Sup** |
| Protein coding | 1 | 48,185,702 | 39,657,575 | 12,877,420 |  | 7249 | 13,404 | 4677 |
|  | 2 | 45,108,953 | 36,583,300 | 40,756,487 |  | 10,916 | 13,349 | 7603 |
|  | 3 | 84,653,437 | 35,192,509 | 38,799,786 |  | 10,332 | 13,287 | 8306 |
| lincRNA | 1 | 1,605,568 | 2,258,887 | 465,116 |  | 167 | 1479 | 115 |
|  | 2 | 1,099,951 | 1,908,176 | 1,431,990 |  | 573 | 1399 | 247 |
|  | 3 | 1,253,235 | 2,202,160 | 1,573,973 |  | 503 | 1399 | 324 |
| Pseudogene | 1 | 1,345,890 | 689,139 | 510,481 |  | 741 | 3454 | 705 |
|  | 2 | 1,497,571 | 618,237 | 1,361,392 |  | 1598 | 3277 | 1224 |
|  | 3 | 2,876,392 | 647,515 | 1,389,824 |  | 1488 | 3228 | 1404 |
| misc RNA | 1 | 47,942 | 1,217,912 | 26,684 |  | 23 | 309 | 31 |
|  | 2 | 29,145 | 1,112,334 | 115,942 |  | 64 | 281 | 55 |
|  | 3 | 48,133 | 852,753 | 98,733 |  | 67 | 281 | 67 |
| Antisense | 1 | 60,242 | 555,246 | 24,972 |  | 144 | 2063 | 91 |
|  | 2 | 60,243 | 486,191 | 101,700 |  | 644 | 2028 | 234 |
|  | 3 | 92,719 | 415,023 | 78,957 |  | 555 | 1968 | 318 |
| Mt tRNA | 1 | 614,877 | 14,759 | 43,723 |  | 22 | 21 | 21 |
|  | 2 | 448,917 | 13,463 | 119,988 |  | 21 | 20 | 21 |
|  | 3 | 862,989 | 18,251 | 110,358 |  | 21 | 20 | 21 |
| Processed transcript | 1 | 55,422 | 115,917 | 17,342 |  | 52 | 234 | 40 |
|  | 2 | 40,117 | 100,741 | 58,974 |  | 125 | 231 | 78 |
|  | 3 | 65,060 | 98,190 | 50,627 |  | 109 | 218 | 76 |
| Sense intronic | 1 | 11,667 | 52,328 | 12,737 |  | 34 | 374 | 34 |
|  | 2 | 12,969 | 48,063 | 36,988 |  | 144 | 355 | 72 |
|  | 3 | 21,171 | 46,772 | 34,796 |  | 121 | 358 | 98 |
| circRNA | 1 | 28,972 | 97,606 | 22,804 |  | 102 | 1530 | 88 |
|  | 2 | 17,944 | 94,606 | 70,070 |  | 513 | 1483 | 315 |
|  | 3 | 40,018 | 88,644 | 59,608 |  | 537 | 1366 | 410 |
| snRNA | 1 | 2430 | 28,533 | 1039 |  | 19 | 257 | 20 |
|  | 2 | 1138 | 25,687 | 5105 |  | 49 | 238 | 59 |
|  | 3 | 2242 | 26,694 | 4066 |  | 59 | 242 | 63 |
| Sense overlapping | 1 | 6709 | 16,113 | 2546 |  | 16 | 99 | 10 |
|  | 2 | 6685 | 15,016 | 11,330 |  | 49 | 89 | 31 |
|  | 3 | 10,955 | 14,611 | 7479 |  | 43 | 95 | 26 |
| Mt rRNA | 1 | 14,368 | 1024 | 8715 |  | 2 | 2 | 2 |
|  | 2 | 10,758 | 221 | 12,291 |  | 2 | 2 | 2 |
|  | 3 | 24,559 | 559 | 12,054 |  | 2 | 2 | 2 |
| snoRNA | 1 | 2125 | 20,585 | 3348 |  | 15 | 281 | 12 |
|  | 2 | 2200 | 18,768 | 5334 |  | 67 | 275 | 41 |
|  | 3 | 4053 | 17,991 | 5742 |  | 78 | 255 | 59 |
| 3' overlapping ncRNA | 1 | 1305 | 3233 | 1082 |  | 3 | 8 | 3 |
|  | 2 | 1082 | 3153 | 2111 |  | 7 | 7 | 2 |
|  | 3 | 3374 | 3028 | 2427 |  | 3 | 8 | 6 |
| miRNA | 1 | 729 | 2789 | 373 |  | 7 | 91 | 8 |
|  | 2 | 741 | 2569 | 1002 |  | 41 | 80 | 19 |
|  | 3 | 953 | 2539 | 1024 |  | 35 | 90 | 18 |
| Others | 1 | 477 | 846 | 0 |  | 3 | 13 | 0 |
|  | 2 | 278 | 781 | 366 |  | 6 | 14 | 2 |
|  | 3 | 362 | 616 | 539 |  | 3 | 10 | 3 |
| rRNA | 1 | 105 | 363 | 295 |  | 1 | 14 | 3 |
|  | 2 | 52 | 339 | 128 |  | 2 | 13 | 3 |
|  | 3 | 59 | 308 | 152 |  | 4 | 13 | 5 |

*Note*: SG, stress granules.
