# Supplementary material for: Screening Linear and Circular RNA Transcripts from Stress Granules
Source: Genomics Proteomics Bioinformatics. 2022 Jan 25;21(4):886–93. doi: 10.1016/j.gpb.2022.01.003 (PMC10787114; doi:10.1016/j.gpb.2022.01.003)
Supplement: Supplementary Table S2 [file mmc3.docx]

**Table S2 Information and translation potential of SG-enriched circRNAs**

| **circRNA location** | **circAtlas ID** | **Length (nt)** | **GC (%)** | **m^6^A sites** | **RP/PP** |
| --- | --- | --- | --- | --- | --- |
| chr2:109369454\|109389502 | hsa-RANBP2_0014 | 341 | 34.90 | 1 | 0.3 |
| chr12:88481557\|88524995 | hsa-CEP290_0015 | 75 | 36.00 | 1 | 0.3 |
| chr4:1902353\|1936989 | hsa-WHSC1_0004 | 1057 | 48.91 | 0 | 0.3 |
| chr11:129996595\|130005610 | hsa-APLP2_0001 | 459 | 51.63 | 1 | 0.3 |
| chr20:61522312\|61545758 | hsa-DIDO1_0008 | 637 | 49.61 | 1 | 0 |
| chrX:73812178\|73815835 | hsa-RLIM_0001 | 192 | 40.62 | 0 | 0 |
| chr1:76253182\|76259918 | hsa-RABGGTB_0005 | 150 | 42.67 | 0 | 0 |
| chr2:191300703\|191302287 | hsa-MFSD6_0001 | 1585 | 45.55 | 1 | 0 |
| chr3:188326949\|188426181 | hsa-LPP_0001 | 127 | 52.76 | 1 | 0 |
| chr2:234164748\|234178713 | hsa-ATG16L1_0003 | 160 | 48.75 | 1 | 0 |
| chr4:7774547\|7780603 | hsa-AFAP1_0006 | 471 | 59.66 | 1 | 0 |
| chr2:97856715\|97877478 | hsa-ANKRD36_0001 | 320 | 42.50 | 0 | 0.3 |
| chrX:130870155\|130928494 | hsa-FIRRE_0016 | 170 | 44.12 | 0 | 0.3 |
| chr15:59204762\|59209198 | hsa-SLTM_0001 | 391 | 37.85 | 0 | 0.3 |
| chr3:27478879\|27490288 | hsa-SLC4A7_0048 | 286 | 41.96 | 0 | 0.3 |
| chr1:7837220\|7838229 | hsa-VAMP3_0001 | 262 | 48.47 | 0 | 0.3 |
| chr1:35643590\|35653691 | hsa-SFPQ_0006 | 342 | 48.54 | 0 | 0.3 |
| chr2:72958136\|72960247 | hsa-EXOC6B_0010 | 412 | 35.92 | 0 | 0.3 |
| chr11:103013997\|103093815 | hsa-DYNC2H1_0065 | 374 | 38.50 | 0 | 0 |
| chr18:8718422\|8720494 | hsa-MTCL1_0002 | 388 | 48.97 | 0 | 0.3 |
| chr1:245017620\|245026032 | hsa-HNRNPU_0070 | 186 | 38.17 | 0 | 0 |
| chr7:27582586\|27689252 | hsa-HIBADH_0005 | 405 | 46.67 | 0 | 0 |
| chr17:16046920\|16052831 | hsa-NCOR1_0033 | 331 | 39.88 | 0 | 0 |
| chr12:22622643\|22659753 | hsa-C2CD5_0014 | 925 | 40.76 | 0 | 0 |
| chr15:42736400\|42738856 | hsa-ZNF106_0020 | 131 | 36.64 | 0 | 0 |
| chr15:93496587\|93524687 | hsa-CHD2_0130 | 190 | 38.95 | 0 | 0 |
| chr6:44364078\|44366664 | hsa-CDC5L_0025 | 100 | 46.00 | 0 | 0 |
| chr10:75299202\|75336119 | hsa-USP54_0076 | 1942 | 44.54 | 0 | 0 |
| chr5:122682299\|122718540 | hsa-CEP120_0005 | 203 | 41.38 | 0 | 0 |
| chr5:74698797\|74706928 | hsa-COL4A3BP_0027 | 222 | 41.89 | 0 | 0 |
| chr5:41749629\|41807540 | hsa-OXCT1_0028 | 687 | 43.09 | 0 | 0 |
| chr5:133493398\|133510205 | hsa-SKP1_0028 | 53 | 35.85 | 0 | 0 |
| chr2:85595809\|85604597 | hsa-ELMOD3_0001 | 415 | 57.35 | 0 | 0 |
| chr10:70670063\|70679737 | hsa-DDX50_0008 | 226 | 42.48 | 0 | 0 |
| chr5:40746900\|40747121 | hsa-TTC33_0003 | 222 | 43.69 | 0 | 0 |
| chr7:134632259\|134635265 | hsa-CALD1_0004 | 403 | 55.83 | 0 | 0 |
| chr14:39870380\|39871715 | hsa-FBXO33_0002 | 111 | 37.84 | 0 | 0 |
| chr15:91503130\|91505004 | hsa-RCCD1_0001 | 457 | 56.89 | 0 | 0 |
| chr3:44986660\|45000952 | hsa-ZDHHC3_0001 | 557 | 54.76 | 0 | 0 |
| chr10:131334505\|131445098 | hsa-MGMT_0013 | 1308 | 49.24 | 0 | 0 |
| chr18:12546673\|12546903 | hsa-SPIRE1_0010 | 231 | 43.29 | 0 | 0 |
| chr15:40901045\|40911209 | hsa-CASC5_0012 | 380 | 39.47 | 0 | 0 |
| chr21:33979959\|33982315 | hsa-C21orf59_0001 | 313 | 43.45 | 0 | 0 |
| chr10:15838099\|15879317 | hsa-FAM188A_0040 | 339 | 38.35 | 0 | 0 |
| chr14:90446884\|90451554 | hsa-TDP1_0014 | 340 | 43.53 | 0 | 0 |
| chr12:112515992\|112516545 | hsa-NAA25_0020 | 554 | 38.09 | 0 | 0 |
| chr11:117206321\|117209384 | hsa-CEP164_0007 | 179 | 46.93 | 0 | 0 |
| chrX:12817326\|12828265 | hsa-PRPS2_0001 | 408 | 50.25 | 0 | 0 |
| chr18:60608951\|60630705 | hsa-PHLPP1_0001 | 437 | 47.83 | 0 | 0 |
| chr11:65272291\|65272490 | hsa-MALAT1_0018 | 200 | 40.50 | 0 | 0 |
| chr2:114688878\|114699936 | hsa-ACTR3_0011 | 174 | 34.48 | 0 | 0 |
| chr2:159477502\|159477933 | hsa-PKP4_0001 | 323 | 45.82 | 0 | 0 |
| chr10:22856780\|22880710 | hsa-PIP4K2A_0003 | 381 | 45.67 | 0 | 0 |
| chr2:26346852\|26350820 | hsa-RAB10_0009 | 257 | 38.13 | 0 | 0 |
| chr16:10508431\|10534319 | hsa-ATF7IP2_0024 | 455 | 40.66 | 1 | 0 |
| chr2:135624112\|135626611 | hsa-CCNT2-AS1_0003 | 1353 | 44.27 | 0 | 0.3 |
| chr9:100773595\|100774754 | hsa-ANP32B_0007 | 129 | 41.86 | 0 | 0 |
| chr2:214012432\|214014971 | hsa-IKZF2_0007 | 221 | 48.42 | 0 | 0 |
| chr1:1586823\|1650894 | hsa-RP1-283E3_0030 | 214 | 44.86 | NA | NA |
| chr1:14095613\|14109326 | hsa-PRDM2_0004 | 167 | 47.31 | NA | NA |
| chr18:42363353\|42383064 | hsa-SETBP1_0015 | 140 | 36.43 | NA | NA |
| chr15:68476357\|68479011 | hsa-PIAS1_0040 | 979 | 41.06 | NA | NA |
| chr19:47421745\|47492932 | hsa-CTD-2233K9_0001 | 355 | 50.42 | NA | NA |
| chr14:32559708\|32563592 | hsa-ARHGAP5_0003 | 545 | 37.06 | NA | NA |
| chr10:64936057\|64937616 | hsa-JMJD1C_0015 | 669 | 38.42 | NA | NA |
| chr6:90959408\|90962309 | hsa-BACH2_0013 | 246 | 44.31 | NA | NA |

*Note*: RP/PP, ribosome profiling and polysome profiling evidence.
