# Supplementary material for: Screening Linear and Circular RNA Transcripts from Stress Granules
Source: Genomics Proteomics Bioinformatics. 2022 Jan 25;21(4):886–93. doi: 10.1016/j.gpb.2022.01.003 (PMC10787114; doi:10.1016/j.gpb.2022.01.003)
Supplement: Supplementary Table S3 [file mmc4.docx]

**Table S3 DNA Oligos used in this study**

| **Oligo name** | **Sequence** |
| --- | --- |
| T7- circARHGAP5-prob-F | TGTAATACGACTCACTATAGGGGATCATCTTCCTTTTTATCT |
| T7- circARHGAP5-prob-R | AGATAAAAAGGAAGATGATCCCCTATAGTGAGTCGTATTACA |
| T7- circSLTM-prob-F | TGTAATACGACTCACTATAGGGTGTTTTTTACTTGGGCTGTT |
| T7- circSLTM-prob-R | AACAGCCCAAGTAAAAAACACCCTATAGTGAGTCGTATTACA |
| T7- COL7A1-prob-F | TGTAATACGACTCACTATAGGGTCTCCAACTTCACCATCTC |
| T7- COL7A1-prob-R | GAGATGGTGAAGTTGGAGACCCTATAGTGAGTCGTATTACA |
| T7- ANKRD11-prob-F | TGTAATACGACTCACTATAGGGGGGCTGTATATATTATGTC |
| T7- ANKRD11-prob-R | GACATAATATATACAGCCCCCCTATAGTGAGTCGTATTACA |
