# Supplementary material for: Screening Linear and Circular RNA Transcripts from Stress Granules
Source: Genomics Proteomics Bioinformatics. 2022 Jan 25;21(4):886–93. doi: 10.1016/j.gpb.2022.01.003 (PMC10787114; doi:10.1016/j.gpb.2022.01.003)
Supplement: Supplementary Table S4 [file mmc5.docx]

**Table S4 Antibodies used in this study**

| **Antibody name** | **Company** | **Catalog number** |
| --- | --- | --- |
| G3BP1 | Proteintech | 13057-2-AP |
| G3BP1 | Proteintech | 66486-1-AP |
| GAPDH | EASYBIO | BE0023 |
| Anti-Mouse IgG-HRP conjugated | EASYBIO | BE0102 |
| Anti-Rabbit IgG-HRP conjugated | EASYBIO | BE0101 |
| Anti-Rabbit IgG (Alexa Fluor 555 conjugate) | Cell Signaling Technology | ＃4413 |
| Anti-Biotin (FITC conjugate) | abcam | ab6650 |
